# Post-Voided Residual Ratio Does Not Predict Trifecta Outcome after Transurethral Resection of Prostate

**DOI:** 10.3390/life14040445

**Published:** 2024-03-27

**Authors:** Riccardo Lombardo, Nicola Ghezzo, Luca Sarcinelli, Beatrice Turchi, Filippo Zammitti, Antonio Franco, Antonio Nacchia, Antonio Cicione, Giorgia Tema, Antonio Luigi Pastore, Giorgio Guarnotta, Andrea Fuschi, Yazan Al Salhi, Andrea Tubaro, Cosimo De Nunzio

**Affiliations:** Department of Urology, Sapienza University of Rome, 00185 Rome, Italy; nicola.ghezzo@uniroma1.it (N.G.); luca.sarcinelli@uniroma1.it (L.S.); beatrice.turchi@uniroma1.it (B.T.); filippo.zammitti@uniroma1.it (F.Z.); antonio.franco@uniroma1.it (A.F.); antonio.nacchia@uniroma1.it (A.N.); antonio.cicione@uniroma1.it (A.C.); giorgia.tema@uniroma1.it (G.T.); antonioluigi.pastore@uniroma1.it (A.L.P.); giorgio.guarnotta@uniroma1.it (G.G.); andrea.fuschi@uniroma1.it (A.F.); yazan.alsalhi@uniroma1.it (Y.A.S.); andrea.tubaro@uniroma1.it (A.T.); cosimo.denunzio@uniroma1.it (C.D.N.)

**Keywords:** TURP, IPSS, outcomes, post-voided residual, LUTS, BPH

## Abstract

The purpose of this study was to assess the importance of the post-void residual (PVR) ratio (PVR ratio) in achieving a favorable trifecta outcome for patients suffering from lower urinary tract symptoms and benign prostatic enlargement (LUTS-BPE) who undergo transurethral resection of the prostate (TURP). Starting from 2015, a series of patients with LUTS-BPE who underwent TURP were included in a forward-looking study. These patients were assessed using the international prostate symptom score (IPSS) screening tool, uroflowmetry, and a transrectal ultrasound to measure prostate volume (TRUS). Both the PVR urine volume and the PVR ratio (PVR-R), which is the PVR as a percentage of total bladder volume (voided volume + PVR), were measured. The assessment of outcomes was based on the trifecta favorable outcome, defined as meeting all of the following criteria: (1) absence of perioperative complications, (2) a postoperative IPSS of less than eight, and (3) a postoperative maximum urinary flow rate (Qmax) greater than 15 mL/s. A total of 143 patients were included, with a median age of 70 years (interquartile range 65–73). Of these, 58% (83/143) achieved a positive trifecta outcome. Upon conducting a multivariate analysis, both IPSS and Qmax were identified as predictors of a positive trifecta outcome, whereas the PVR-R did not prove to be an independent predictor. In summary, it was found that preoperative IPSS and Qmax are indicative of a trifecta outcome following TURP, whereas PVR-R is not.

## 1. Introduction

Benign prostatic hyperplasia (BPH) results in a benign enlargement of the prostate (BPE), leading to lower urinary tract symptoms (LUTS). These symptoms can be distressing and lead to substantial morbidity [1]. The gold standard surgical treatment for relieving bladder outlet obstruction (BOO) in patients with LUTS by BPE is transurethral resection of the prostate (TURP) [2].

However, approximately 25–30% of patients express dissatisfaction after undergoing TURP. Research has revealed that both subjective and objective failures are linked to detrusor dysfunction, encompassing conditions of overactivity or underactivity, and ambiguous bladder outlet obstruction (BOO), rather than the recurrence of benign prostatic obstruction (BPO) [3,4,5].

Although many definitions of successful outcome exist in the literature, most of the papers define it as an improvement from baseline in international prostate symptom score (IPSS) and maximum urinary flow (Qmax) values [6]. Nowadays, urodynamic investigations with pressure flow studies (PFSs) represent the gold standard for diagnosis of BPO and predicting TURP outcomes [7]. Many efforts have been made in order to develop new non-invasive methods in order to predict BOO, and consequently TURP, outcomes.

Parameters of a prostate ultrasound scan, including transitional zone (TZ), prostatic volume, prostate shape, intra-prostatic protrusion (IPP), bladder/detrusor wall thickness (DWT) or weight (UEBW), and other non-invasive pressure flow tests have been widely evaluated. However, all of these methods are still considered investigational [8,9,10].

Post-voided residual (PVR) is a recommended method to evaluate bladder emptying and is measured by transabdominal ultrasound, bladder scan, or catheterization [11]. Increased PVR volumes contribute to chronic bladder distension, diminished bladder compliance, and hypotonia, which in turn result in compromised detrusor muscle function and less favorable outcomes after surgery. Suhani and colleagues have shown that patients with a PVR of less than 60 mL who underwent surgery experienced improved post-operative outcomes, as evidenced by higher maximum and average flow rates, along with better scores on the IPSS, both objectively and subjectively [6,12].

Nonetheless, this straightforward technique has two primary drawbacks. Firstly, it is unable to distinguish between BOO/BPH and detrusor underactivity (DUA), or determine if both conditions are present. Additionally, there is currently no established standard volume for PVR that can be used for pathological referrals [13].

In order to overcome these limitations, some authors have proposed different parameters that provide a more accurate assessment of bladder emptying effectiveness or impairment when compared to PVR. These parameters include bladder voiding efficiency (BVE) and PVR-R, which offer more precise insights [14].

Since PVR-R has been poorly evaluated in patients undergoing TURP, the primary aim of our study was to evaluate the role of the PVR-R in predicting a favorable trifecta outcome among patients with LUTS-BPE who undergo TURP.

## 2. Materials and Methods

Beginning in 2021, a sequential cohort of patients with lower urinary tract symptoms due to benign prostatic enlargement (LUTS-BPE) who underwent transurethral resection of the prostate (TURP) were systematically registered for this study. This investigation entails a retrospective review of data collected in a forward-looking manner. Exclusion criteria included patients with neurological disorders, renal insufficiency, bladder stones, prostate cancer, urethral stricture, and those who had undergone prior pelvic surgery. The research adhered to the ethical guidelines outlined in the Declaration of Helsinki. The study was approved by the ethical board (Comitato Etico Università Sapienza-Prot. 258 SA_2021 RIF. CE 6376_2021), and all patients signed dedicated informed consent granting permission to use their clinical parameters for research projects.

A comprehensive clinical assessment was conducted for all patients, encompassing a thorough collection of their medical history and a physical examination that included a digital rectal examination (DRE) and a transrectal ultrasound prostate volume assessment (TRUS). Ultrasound measurements were carried out using a 3.5-MHz convex probe. LUTS were evaluated using the IPSS, which also included a question related to the patient’s quality of life. Additionally, all patients underwent free uroflowmetry, PVR urine, and the percentage of PVR to bladder volume (BV) measurement.

Every participant conducted a minimum of two free-flow tests to achieve a voided volume of at least 150 mL. The instance of best voiding performance from these tests was chosen for the purpose of statistical evaluation. The peak flow rate, as automatically determined by the flowmeter, was utilized for analysis. In cases where artifacts affected the measurement, the 2-second rule was applied to adjust for these inaccuracies. Following these tests, the volume of residual urine was assessed using suprapubic ultrasound.

PVR-R = PVR/BV. Bladder Volume = VV + PVR. PVR-R = (PVR/total BV) × 100. All ultrasound measurements were performed by two certified urologists with more than 10 years of experience (RL and AC).

Outcomes were assessed three months post-operation based on the criteria for a favorable trifecta outcome, which is determined by achieving all of the following: (1) the absence of perioperative complications, (2) a postoperative IPSS of less than eight, and (3) a postoperative maximum urinary flow rate (Qmax) greater than 15 mL/s.

Statistical analysis was performed using the Statistical Package for the Social Sciences (SPSS v.24, IBM Corp., Armonk, NY, USA) and STATA software (version 14). The analysis of the data distribution using the Kolmogorov-Smirnov test revealed that the dataset for the study was not normally distributed. To compare groups of patients, the medians for quantitative variables were examined using the Kruskal-Wallis one-way analysis of variance, while the Chi-squared test was applied to assess differences in distributions for categorical variables. Initially, all variables were evaluated through univariate binary logistic regression to predict bladder outlet obstruction (BOO). Subsequently, those variables that were statistically significant were incorporated into a multivariable logistic regression model adjusted for age.

## 3. Results

Overall, 143 patients were enrolled, with a median age of 70 years (IQR 65/73) and a median BMI of 25 kg/m^2^ (IQR: 24/28). The enrolled cohort was symptomatic (median IPSS 17: IQR 13/23) and exhibited poor flow (median Qmax 8: IQR 6/10.85). On ultrasound, the median PV was 60 (IQR 46.5/84.5). The median PVR-R was 12% (IQR 7/23). Characteristics of the enrolled population are listed in Table 1.

Positive trifecta outcome was observed in 58% (83/143) of the patients. Successful outcomes were detected in patients with lower preoperative IPSS (median of 16 (13/20) vs. 19 (14/24): *p* value < 0.005) and higher preoperative Qmax (median of 9 (6.3/12) vs. 7.3 (6/8.8): *p* value < 0.005). No statistically significant differences were recorded regarding age, BMI, and PV when comparing patients with successful or unsuccessful trifecta outcome (*p* > 0.05). Neither PVR ratio presented significant difference between two groups (median of 13% (8/24) vs. 11% (5/22): *p* = 0.53) (Table 2).

In the univariate binary logistic regression, preoperative IPSS Score (OR 0.94: *p* value = 0.025) and preoperative Qmax (OR 1.21: *p* value = 0.002) were predictors of positive trifecta outcomes after the intervention. The PVR ratio was not shown to be an outcome predictor (OR 1.11: *p* value = 0.93).

In the multivariable logistic regression model, IPSS (odds ratio [OR] 0.94 per point; 95% confidence interval [CI]. 0.88–0.99; *p* value = 0.046) and Qmax (odds ratio [OR] 1.26 per mL/s; 95% confidence interval [CI]. 1.10–1.44; *p* value = 0.001) were shown to be predictors of a positive trifecta outcome (Table 3).

## 4. Discussion

In this study we evaluated PVR-R as a possible predictor of favorable outcome in patients undergoing TURP. According to our results, patients with a low preoperative IPSS and a high preoperative Qmax have increased odds of achieving a positive trifecta outcome. However, there was not a role for PVR-R in predicting positive outcomes after TURP.

In the past years, several non-invasive methods have been investigated for predicting TURP outcome. Shinbo et al. evaluated the usefulness of preoperative IPSS and several TRUS parameters in predicting TURP outcome in a prospective cohort of 560 patients. They observed an area under the ROC curve of 0.663 (95% CI: 0.621–0.705) for preoperative IPSS, 0.691 (95% CI: 0.650–0.731) for TPV, 0.719 (95% CI: 0.678–0.757) for TZ index, and 0.845 (95% CI: 0.811–0.875) for resistive index (RI) [9]. Overall, Shinbo’s cohort evaluated a similar population of symptomatic patients with a high median age undergoing TURP. Our results are in line with Shinbo’s results and confirm the important role of preoperative characteristics in predicting TURP outcomes. Moreover, Ahyai et al. published a meta-analysis on 20 contemporary RCTs published between 2005 and 2009 with an overall sample size of 954 TURP patients and a maximum follow-up of 5 years to evaluate TURP outcome [15]. Our results are in line with Ahyai’s meta-analysis, with TURP resulting in a substantial improvement in mean Qmax (+162%) and a significant reduction in mean IPSS (−70%), mean QoL scores (−69%), and mean PVRU (−77%), and with the results gained by Seki et al., showing that the decrease in total IPSS averaged 11.9, while the increase in Qmax was 8.1 (mL/s) at 12 months after treatment. Our cohort clearly differs from some of the studies included in the meta-analysis, considering we enrolled a consecutive series of older (median age = 70) and symptomatic (median IPSS = 16) patients, explaining some small differences in terms of treatment outcomes.

Hakenberg et al., in their prospective analysis of the relevant variables for TURP outcome, showed how only age can be considered a significant risk factor for poor TURP outcome in multivariate analysis (*p* < 0.05) [16]. However, the Hakenberg study was published almost 30 years ago, and focused mainly on IPSS and uroflowmetry without a specific focus on ultrasound measurements.

Recently, we developed the Young Academic Urologist nomogram for the prediction of TURP outcome, which showed a 6% better chance of good TURP outcome per point of preoperative IPSS score, and a 5% better chance of having a good outcome on TURP per year of younger age [17]. The innovative Young Academic Urologist nomogram demonstrated an area under the curve (AUC) of 0.77, with a 95% confidence interval (CI) ranging from 0.70 to 0.83, for predicting outcomes of transurethral resection of the prostate (TURP). At an optimal threshold of 75% probability according to the nomogram, the sensitivity was 62% and the specificity was 73%. Furthermore, the positive predictive value (PPV) was 81%, while the negative predictive value (NPV) stood at 52%.

Some authors [18,19,20] have recently introduced two important parameters to research in this area: bladder voiding efficiency (BVE), and PVR-R. BVE is defined as the product of bladder contractility against urethral resistance, measured according to the degree of bladder emptying, and defined as (voided volume/total bladder capacity) × 100. It is a parameter that specifically considers the voided volume, providing insight into the bladder’s contractile function and reflecting the success rate of bladder emptying. The PVR-R is a measure of a bladder emptying failure, centered on the PVR and defined by PVR/total bladder capacity. This parameter reflects the dysfunctional storage of urine in the bladder following urination and is more closely associated with voiding/emptying than the PVR volume itself, making it a more reliable measure of the extent of bladder emptying failure. While the BVE reflects the bladder’s contractile function, PVR-R measures the deficiency in bladder emptying. Therefore, BVE and PVR-R are contrasting parameters and should not be considered as equivalent expressions of bladder emptying measurement. Both BVE and PVR-R are valuable tools that contribute to a comprehensive understanding of bladder voiding function from various perspectives.

In this study we explored PVR-R, which takes into consideration both the total bladder capacity and total voided volume. The incorporation of these factors into PVR-R makes it potentially more effective in identifying and accurately assessing bladder emptying impairment. Additionally, PVR-R allows for better comparability among different patients.

Rubilotta and colleagues explored the significance of PVR-R in 410 patients undergoing pressure-flow studies (PFS) for lower urinary tract symptoms (LUTS) [13]. Their findings indicated that the post-void residual ratio (PVR-R) was notably elevated in patients with bladder outlet obstruction (BOO), regardless of the state of detrusor muscle contractility. Applying a threshold of 20% for the PVR ratio yielded a positive predictive value (PPV) of 85% for identifying patients with BOO who have normal detrusor contractility [21].

Using a 40% cut-off value, PVR-R demonstrated strong reliability in identifying males who may be at risk of developing obstruction and detrusor contractility disorders, as well as experiencing more severe voiding dysfunction. This risk increases as the PVR-R rises. Therefore, in the case of males being investigated for LUTS, those with elevated PVR-R values (>40%) should be considered as “red flag patients” due to their significantly heightened risk of having the most impaired bladder emptying condition. Rubilotta’s cohort enrolled a younger population (median age 61 years) and performed a dichotomic analysis; therefore, a direct comparison with the present study is difficult.

In our analysis, PVR-R poorly predicted TURP outcomes. According to our results, only higher Qmax and lower IPSS are related to positive outcomes after TURP, while other patient characteristics, such as age, BMI, and PV, have no prognostic value. Our results are in contrast with the conclusions reached by Huang et al. and Seki et al., which showed how patients with lower preoperative IPSS are at increased risk of poor TURP outcome (IPSS OR = 0.13, 95% CI: 0.02–0.95, and IPSS OR = 0.94, 95% CI: 0.88–0.99, respectively) [3,10]. These differences clearly depend on the decision to apply a cut off instead of a variation from baseline to define a positive outcome as described by Suhani et al. [6]. More specifically, the authors used improvements, while we used cut-off, as a proxy of success.

The abovementioned literature opens an important debate on the ideal proxy to evaluate TURP outcomes. As highlighted earlier, several studies with different definitions have evaluated predictors of TURP outcomes. The heterogeneity of the definitions represents a major bias in the comparison of these studies. In recent years, special attention has been drawn to patient reported outcomes, which is not considered a proxy of TURP outcome in the available literature. It is likely that a Qmax cut-off or an IPSS cut-off is not representative of a good outcome, and future studies should assess predictors of patient satisfaction or regret to really identify those patients who will truly benefit from TURP. Alternatively, international guidelines should clearly establish specific cut-offs to define a successful TURP.

Furthermore, it appears fundamental to evaluate the baseline condition of the patient’s bladder to gain a more precise prediction of positive or negative outcomes after unobstructive treatment, offering the patients better counseling. DO, defined as spontaneous or provoked involuntary detrusor contractions during the filling phase, and DUA, defined as contractions of reduced strength and/or duration resulting in prolonged bladder emptying and/or failure to achieve complete bladder emptying within a normal time span, are frequently observed in LUTS/BPH patients in combination with BPO or otherwise [22]. Urodynamic studies have revealed DO and DUA in 31% to 68% and in 11% to 40% of patients with LUTS/BPH, respectively. Moreover, both dysfunctions have been reported to coexist in 32.1% of patients with LUTS/BPH [20]. Studies have shown that increased bladder pressures, such as those observed with BPO, can cause morphological and functional remodeling within the various cellular compartments of the bladder wall. The mechanisms that may potentially cause DO in subjects with BPO include: partial denervation of the detrusor muscle resulting in postjunctional supersensitivity, detrusor hypoxia, and abnormal sensory stimuli deriving from anatomical alterations of the prostatic urethra [23]. On the other hand, increased bladder pressure may cause collagen accumulation within the detrusor muscle and subsequent impairment of bladder contractility [24]. However, both DO and DUA may also be related to other factors, including ageing. Indeed, the prevalence of both conditions increases with ageing in men not suffering from obstruction. The presence of DUA and/or DO in LUTS/BPH patients is recognized as an important issue, mainly when selecting and counseling patients for surgical interventions. Indeed, although treatments aimed at relieving BPO have the potential to improve both contractility and instability and prevent the progression of bladder dysfunctions, the persistence of DO and/or DUA may impair surgical outcomes and cause surgical failures [25]. Given the progressive ageing of the male population, the increasing prevalence of diabetes and neurological diseases, as well as progressive delays in LUTS/BPH surgery, a growing prevalence of DO and DUA is expected to occur in the coming years, and the issue of their impact on surgical outcomes will garner more and more research interest [26].

Indeed, persistence of these dysfunctions might be accountable for poor surgical outcomes or surgical failures despite de-obstruction. In detail, persistent DUA may be responsible for postoperative inability to void with need for long-term intermittent catheterization, and for this reason, it has been controversial for many years as to whether surgery for LUTS/BPH should be contraindicated in patients with DUA [8]. On the other hand, persistence of DO may be responsible for a lack of improvement in terms of storage LUTS and postoperative urge urinary incontinence. To date, invasive UDS represents the only reliable method to diagnose these conditions [21]. The persistence of DO and DUA despite surgery may be related to irreversible bladder damage due to chronic obstruction or due to a primary disorder of the bladder that has no relation with BPO [27].

Surgical de-obstruction of the lower urinary tract has been shown to improve urodynamic and clinical parameters in a substantial percentage of patients with DUA and DO at short- and medium-term follow-ups. Indeed, surgery may improve bladder contractility in patients with DUA and a significant percentage of them can recover the ability to void spontaneously. Moreover, surgery for LUTS/BPH may lead to a resolution of DO [28].

In patients with DUA, the reduction of bladder outlet resistance can allow improved bladder emptying through both easier abdominal straining and improved bladder contractility [28]. The increase in bladder contractility can be attributable to improvement of bladder blood flow and cellular function, thanks to decreased bladder pressure and to the destruction of sympathetic adrenergic nerves inhibiting detrusor contractility, located within the bladder neck and prostatic urethra [23]. On the other hand, potential mechanisms behind the improvement of DO following surgery include detrusor re-innervation, permanent surgical ablation of sensory stimuli from the prostatic urethra, PVR reduction, and a subsequent increase in time for bladder filling [21].

Two recent meta-analyses have been published by Kim et al. in which the authors evaluated the impact of preoperative DUA or DO in LUTS/BPH patients undergoing de-obstructive surgery, based on results from comparative studies [29,30]. The authors found that preoperative DUA was significantly associated with poorer degrees of IPSS and Qmax improvements, as well as a poorer tendency (although not statistically significant) toward IPSS-QoL and PVR improvements. On the other hand, improvements in the total IPSS, IPSS-QoL, Qmax, and PVR were similar between patients with and without preoperative DO [29,30]. Although several studies on the subject have been published, the exact role of DUA and DO before surgical intervention is still to be determined [27,28,31,32,33,34,35,36,37].

We have to acknowledge some limitations to our study. Our outcomes for the prediction of TURP results clearly depend on the enrolled population and cannot be extended to all patients undergoing TURP. Particularly, we excluded LUTS/BPE patients with urinary retention and indwelling catheters, considering that at least one free uroflowmetry is needed in order to assess PVR-R. Our study population is characterized by severe symptoms, smaller prostate sizes, and low residual volumes. An additional potential limitation is that residual volume assessments were conducted using ultrasound (US), instead of through the use of a transurethral catheter.

Furthermore, in our study, we defined positive trifecta outcomes as a combination of the following items: (1) no perioperative complications, (2) postoperative IPSS < 8, and (3) postoperative Qmax > 15 mL/s, as reported in other studies on the same topic, and as defined by Suhani et al. Even if this definition has been widely used in recent literature, it still needs to be further evaluated to reach a spread consensus.

Finally, the lack of long-term follow-up represents another possible limitation. However, evaluation 3 months after surgery is the standard to assess the early outcomes after surgery, as well as any complications. According to the available evidence, only a small number of patients with good results at 1–3 months will fail treatment thereafter.

## 5. Conclusions

PVR-R represents an innovative and precise method for measuring bladder emptying. A heightened PVR-R is associated with more pronounced pathological bladder emptying, particularly in cases where BOO and DUA are present.

Hence, PVR-R could play a significant clinical role in the initial evaluation of males with LUTS, potentially enhancing the identification of patients who would benefit from preoperative UD investigations. By surpassing the limitations of PVR, PVR-R can identify males who might be at a higher risk of voiding dysfunctions.

Despite its important role in preoperative assessment, our work shows that PVR ratio alone does not predict patient outcomes after prostate resection.

## Figures and Tables

**Table 1:** Overall characteristics of the cohort.

	Mean (±SD)	Median (IQR)
Age years	69.0 (±7.5)	70.0 (64.4–73.8)
Prostate Volume mL	66.0 (±27.5)	60.0 (46.5–84.5)
BMI kg/m^2^	25.0 (±3.3)	25.0 (24.0–28.0)
Voiding Score points	9.6 (±4.4)	9.0 (6.0–12.0)
Storage Score points	8.1 (±3.9)	7.0 (5.0–11.0)
IPSS Tot points	17.9 (±6.4)	17.0 (13.0–23.0)
Qmax mL/s	8.7 (±3.3)	8.0 (6.0–10.9)
PVR Ratio %	18.0 (±18.0)	12.0 (7.0–23.0)

**Table 2:** Characteristics and outcomes of patients with successful and unsuccessful outcomes after TURP.

	Unsuccessful	Successful	*p*
Age (years)	71.2 (65.6–75.2)	69.5 (64.2–73.5)	0.170
Prostate Volume (mL)	65.0 (49.0–87.3)	59.0 (44.3–83.0)	0.420
BMI (kg/m^2^)	25.0 (24.0–27.3)	25.0 (24.0–28.0)	0.800
Voiding Score (points)	11.0 (7.0–15.0)	8.0 (6.0–11.0)	0.020
Storage Score (points)	8.0 (5.0–11.0)	7.0 (6.0–10.0)	0.740
IPSS Tot (points)	19.0 (14.0–24.0)	16.0 (13.0–20.0)	0.001 *
Qmax (mL/s)	7.3 (6.0–8.8)	9.0 (6.3–12.0)	0.001 *
PVR Ratio (%)	11.0 (5.0–22.0)	13.0 (8.0–24.0)	0.530

* statistically significant.

**Table 3:** Univariate and multivariate binary logistic regression to predict trifecta outcome.

	Univariate OR	*p*	Multivariate OR	*p*
Age (years)	0.97 (0.92–1.02)	0.258		
Preop IPSS (points)	0.94 (0.89–0.99)	0.025	0.94 (0.88–0.99)	0.046
Prostate Volume (mL)	0.99 (0.98–1.01)	0.528		
Qmax (mL/s)	1.21 (1.07–1.36)	0.002	1.26 (1.10–1.44)	0.001
PVR Ratio (%)	1.11 (0.10–11.9)	0.930		

## Data Availability

Data presented in this study are available on request from the corresponding author.
